# Exploring the Role of Sex and Gender in the Adoption of Assistive Technology in Dementia Care: A Scoping Review

**DOI:** 10.1177/07334648241310708

**Published:** 2025-01-09

**Authors:** Maren Salzwedel, Sytse Zuidema, Helianthe Kort, Sarah Janus

**Affiliations:** 1 University of Groningen, Groningen, the Netherlands; 2 University Medical Center Groningen, Groningen, the Netherlands; 3 University of Applied Sciences Utrecht, Utrecht, the Netherlands; 4 Eindhoven University of Technology, Eindhoven, the Netherlands

**Keywords:** dementia, gender, quality of life, technology

## Abstract

Given that women are disproportionately affected by dementia on several levels and assistive technology (AT) is increasingly used to manage the care of the growing number of people with dementia (PwD), there is an urgent need to understand the role of sex and gender regarding adoption of AT in dementia care. We conducted a scoping review following the framework of Arksey and O’Malley. All identified topics of sex and gender analysis (compatibility, attitude, usage, acceptance, access, usefulness, and well-being) were related to AT adoption, where the majority revealed sex and gender differences. Relevance of topics is discussed in relation to generation, culture, and mental health, including a switch of perspective to the gender of the technology. Even though we demonstrated sex and gender differences in AT adoption, their practical implications need to be further elaborated on in future research.

**What this paper adds**

• This review identifies the influence of sex and gender on the adoption of AT in dementia care from the user’s perspective.
• This review extracted practical implications of sex and gender-specific findings regarding AT in dementia care.

**Applications of study findings**

• To enhance practical usability of the findings, we consulted with an elderly care client representative board to ensure the relevance and completeness of our search strategy in advance.
• All topics of sex and gender analyses were matched to identified barriers and facilitators of adoption of AT and usage, thereby stressing the practical relevance of the role of sex and gender.

Sex and gender differences in the context of dementia are relevant in a variety of topics such as clinical presentation, progression, risk factors (Ferretti et al., 2020; Mielke et al., 2018), research, care technologies, and policies (Castro-Aldrete et al., 2023). Sex refers to specific biological and physiological characteristics (e.g., chromosomes and hormones), whereas gender refers to the socially constructed characteristics that can vary through history and cultures (norms, expectations, behaviors, and roles assigned to a gender; World Health Organization). According to the World Health Organization (2023), women are disproportionately affected by dementia on two levels. First, women are more likely to provide informal care to PwD and are thus at higher risk for experiencing caregiver stress (Derreberry & Holroyd, 2019). Additionally, Alzheimer's disease, the most common form of dementia (World Health Organization, 2023), is more frequently diagnosed in women (Derreberry & Holroyd, 2019). This might be due to sex and gender differences in health-seeking behavior (e.g., women visit their general practitioner more frequently; Ballering et al., 2023), or sex-specific dementia risk factors (e.g., cognitive functioning, genes, and depressive symptoms; Kim et al., 2015). In addition, women with dementia experience more severe and rapid cognitive decline in comparison to men (Laws et al., 2016), and make up the majority of the old population where dementia predominantly occurs (Mielke et al., 2018). Yet, sex and gender has not been considered in the management of dementia to this day (Nebel et al., 2018; Sourial et al., 2020).

To manage the care of the rising number of PwD (i.e., 10 million new cases each year; World Health Organization, 2023), assistive technology (AT) is increasingly promoted (Gathercole et al., 2021; Lariviere et al., 2021). The benefits of AT are recognized in both the home setting (Holthe et al., 2022) and care homes (Dugstad et al., 2019). AT can be used by PwD, informal caregivers (e.g., family or friends), and formal caregivers (Gibson et al., 2015, 2016). AT describes any product (e.g., devices and software), “the primary purpose of which is to maintain or improve an individual’s functioning and independence, and thereby promote their well-being” (World Health Organization, 2022, p. 6). Types of AT in dementia care entail positioning systems, health monitoring systems, coaching systems for both PwD and caregivers, reminder and cognitive support devices, ambient assistive living systems, and multi-sensory environments (Lee-Cheong et al., 2022); telecare, communication aids, electronic games, or reminiscence aids (Gibson et al., 2016); and robots assisting with activities of daily life (Wagner et al., 2022) or social participation (Obayashi et al., 2020).

Regarding the adoption of AT in dementia care (i.e., habitual AT use), research has identified several barriers and facilitators. Barriers include technology anxiety, limited perceived benefits, mismatch between device and needs, resistance to use AT, negative attitude, lack of knowledge, inaccessibility, poor design, and ethical issues. Facilitators contain adaptability, affordability, technology experience, ease of use, increase of independence, enjoyment, safety, and alleviating caregivers stress (Boyle et al., 2022). Notably, the influence of sex and gender on the aforementioned factors is unknown in dementia care. However, individual characteristics such as gender affect some of the abovementioned factors in the general older population (e.g., gender significantly associated with perceived ease of use; Chen & Chan, 2014; men report higher perceived ease of use and technology experience; Heerink, 2011). Furthermore, stereotypical thinking might affect adoption. Older people are subjected to negative stereotypes, prejudice, and discrimination due to ageist beliefs (Burnes et al., 2019), including healthcare (e.g., assuming all older adults are cognitively impaired; Higashi et al., 2012) and technology use (e.g., stereotype threat where older adults refrain from using technology due to their fear of confirming ageist stereotypes; Mariano et al., 2021). Importantly, ageist beliefs intersect with biases toward other marginalized groups (e.g., race and gender; Chrisler et al., 2016). In fact, negative stereotypes linked to older adults (e.g., frailty, weakness, and dependence) are similarly attributed to women (Chrisler et al., 2016). It is likely that the intersection of sexist (e.g., masculinity associated with high technical self-efficacy; Huffman et al., 2013) and ageist stereotypical thinking (older adults are unable to use technology; Mariano et al., 2021) discourages technology use by especially older women significantly.

In fact, not considering sex and gender aspects is thought to hinder the progression of detecting, treating, and managing dementia considerably, thereby denying favorable outcomes for all sexes (Nebel et al., 2018). Acknowledging sex and gender aspects in the development of technologies that will be used for the next decades is inevitable to provide equitable dementia care (e.g., preventing bias and increasing efficiency; Castro-Aldrete et al., 2023). Therefore, the objective of this scoping review is to understand the current role of sex and gender regarding AT adoption in dementia care. Specifically, it will explore the topics of sex and gender analysis and the practical implications of sex and gender-specific results. Thereby, this review will provide guidance on areas of relevance regarding future AT design, implementation, and adoption where the consideration of sex and gender facilitates the tailoring AT to the needs of the dementia care triad.

## Method

This scoping review followed the methodological framework of Arksey and O'Malley (2005) as outlined below, with additional guidance by Peters et al. (2022) for developing the protocol. This review provides an overview of the topics that are analyzed in terms of sex and gender, which is in line with the designated purpose of “summarizing and disseminating research findings” (Arksey & O’Malley, 2005, p. 21). A protocol was developed according to the guidelines of the Joanna Briggs Institute (JBI) and the Preferred Reporting Items for Systematic Reviews and Meta-Analyses: Extension for Scoping Reviews (PRISMA-ScR; Peters et al., 2022) and registered with the Open Science Framework (OSF; https://doi.org/10.17605/OSF.IO/8VKZJ).

### Research Questions

The research questions were developed based on a mnemonic for identifying research questions, which outlines the population, concept, and context of the review (PCC; Peters et al., 2022), which resulted in the following outline: (P) dementia care triad (i.e., PwD, informal caregiver, and professional caregiver) and (C) role of sex and gender factors (C) AT in dementia care setting (community/home setting and care home setting). The research questions this scoping review explored were:

1. What are the topics that are analyzed in terms of sex and gender?
2. What are the sex and gender-specific findings for the different users of the triad?
3. Is there a discussion of the practical implications, if yes: what are the conclusions?

### Consultation

Before running the search, we followed the optional consultation stage described in the framework of Arksey and O'Malley (2005) to assure the accuracy and completeness of our search during this initial phase. Therefore, stakeholders with expertise on the interests of the dementia care triad were contacted via the research institution’s network and consulted (i.e., neuropsychologist in geriatric rehabilitation, nurse in home care, and client council of 17 client representatives of the university network for elderly care of the University Medical Center Groningen). The stakeholders highlighted the importance of social participation, digital technology, meaningful leisure time, communication, and safety. Consequently, social participation and meaningful leisure time were added to the definition of AT and introduction; however, adding terms addressing the aspect of leisure to the search strategy was not possible because this resulted in many irrelevant hits. Robots can enhance communication for PwD (Zhou et al., 2022); therefore, the term “robot” was added to address social participation. Also, we added “digital” to the strategy as suggested by the stakeholders.

### Identifying Relevant Studies

**Figure 1.**
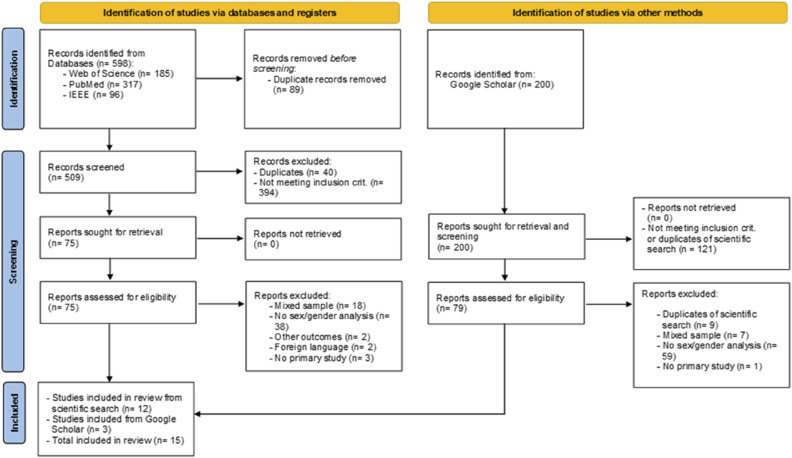
Flow diagram of study selection process.

The search strategy was defined in accordance with the advice of a medical information specialist at the University Medical Library Groningen. The searched databases were PubMed, IEEE Xplore (Institute of Electrical and Electronics Engineers), and Web of Science. Gray literature (i.e., Google Scholar and conference abstracts) was included to capture the yet unpublished literature, thereby accommodating the most recent advances in the field. Prior to translation to the remaining data bases, the search strategy was developed in PubMed as it allows for refinements (e.g., evaluation of search terms). The set-up of the strategy involved the use of Boolean search operators (i.e., AND and OR) and consisted of search terms representing the topics “assistive technology,” “sex/gender,” and “dementia” (Appendix A). AT was defined as technology that addresses PwD or caregiver functioning or health (Lee-Cheong et al., 2022). Acknowledging its heterogeneity, all types will be considered. The terms sex and gender are not currently used according to uniform standards (Adisso et al., 2020), not allowing for their differentiation, which is why this review refers to “sex and gender” overall. No restrictions were made regarding dementia type or publishing year to create a comprehensive overview. The scientific and gray literature searches were conducted on September 20, 2023. One author (MS) removed duplicates manually with reference manager EndNote (The Endnote Team, 2013) before two authors (SJ and MS) independently (i.e., blinding setting) screened title and abstract with the Rayyan tool (Ouzzani et al., 2016). Eligibility screening of the remaining articles was also conducted by two authors (SJ and MS), and before data was charted in Excel by one author (MS). The flowchart below presents a detailed visualization of the screening process (Figure 1).

### Study Selection

Inclusion criteria were (1) AT in line with the definition stated in the method section, (2) used by at least one member of the dementia care triad (i.e., PwD, informal caregiver, or formal caregiver), (3) disaggregation of data by sex or gender, (4) peer-reviewed articles and conference papers, (5) primary studies, and (6) published in English, Dutch, or German in alignment with the languages spoken by the authors. Studies that targeted AT (1) in the context of mild cognitive impairment, (2) for therapeutic purposes, or (3) diagnostic purposes, or (4) only reporting the sex and gender distribution of the sample without analysis, and (5) mixed samples without separate analysis of the dementia care triad, were excluded.

### Data Charting and Reporting the Results

Information relevant to the research questions, as well as general demographics and descriptives were charted in a table format. We conducted thematic inductive analysis to identify the emerging topics that have been analyzed in terms of sex and gender (Research question 1) in both qualitative and quantitative data (Appendix B). Articles were grouped according to the emerging categories for data presentation (Research question 1) and ranked from most to least frequently analyzed. Each topic differentiated between triad member (Research question 2), concluding with practical implications (Research question 3). Data on the above defined outcomes are presented in table form alongside a narrative description. For quantitative studies, this review reported sex and gender-specific findings with general *p*-values due to inconsistent reporting of additional standard statistical metrics among the included studies.

## Results

Fifteen studies were included. A descriptive summary of the results can be found in Table 1, as well as a detailed summary in Table 2. Most studies analyzed AT in general (*n* = 8), while the remaining studies focused on subtypes of AT (*n* = 7). Regarding sex and gender analysis, one study elaborated on sex and gender differences as a main topic (Xiong et al., 2020), whereas the remaining studies conducted side-analyses as part of an independent main topic (*n* = 14). The use of the terms sex and gender differed among the included studies (Table 2). Studies were referring to either gender (*n* = 6) or sex (*n* = 1), or used both terms interchangeably (*n* = 5). Two studies used both terms as an overall concept due to considering them distinct but intertwined. One study described their participants as female without referring to either sex or gender.

**Table 1.** Descriptive Summary of Results With Frequency and Percentages.

Descriptive Variable	*n*	*%*
Triad role		
Informal caregiver	9	60
PwD	8	53.3
Formal caregiver	2	13.3
Region		
Europe	9	60
North America	4	26.6
Asia	2	13.3
Year range		
2005–2010	1	6.6
2011–2015	1	6.6
2016–2020	8	53.3
2021–2023	5	33.3
Topic of analysis		
Compatibility	5	33.3
Attitude	4	26.6
Usage	3	20
Usefulness	3	20
Acceptance	2	13.3
Access	2	13.3
Well-being	2	13.3
AT type		
Multiple types	8	53.3
Commercial technology	1	6.6
Robots	2	13.3
Location monitoring	1	6.6
Telehealth	1	6.6
Digital care platform	1	6.6
Social alarm	1	6.6

*Note.* AT = assistive technology, PwD = person with dementia.

**Table 2.** Summary Table With Demographics, Sex and Gender Analysis and Differences per Topic.

Study	Research Design	Method	Country	AT Type	Triad Member	Term for Concept Sex/Gender	Topic of Sex and Gender Analysis	Sex and Gender Differences
Rialle et al., 2008	Cross-sectional	Questionnaire	France	Surveillance, video-communication, and location monitoring	IC (*n* = 195)	Sex and gender	Attitude	Yes (location monitoring) No (video-communication and surveillance)
Olsson et al., 2011	Cross-sectional	Interview	Sweden	Various types assisting cognition, safety, and caregiving	IC (*n* = 14)	Sex and gender	Compatibility	Yes
Olsson et al., 2016	Repeated interview	Interview	Sweden	Location monitoring	PwD (*n* = 11)	Sex and gender	Compatibility	Yes
O’Sullivan et al., 2017	Cross-sectional	Interview, Questionnaire	Germany	ICT for psychosocial interventions	FC (*n* = 205)	Female	Attitude and compatibility	Attitude: Yes Compatibility: Yes
Xiong, Astell, et al., 2018	Cross-sectional	Questionnaire	Chinese population in Canada	Various types to improve health/psychosocial outcomes	IC (*n* = 40)	Sex and gender	Usefulness	Yes
Asghar et al., 2019	Cross-sectional	Questionnaire	Pakistan	Various types to facilitate care	PwD (*n* = 327)	Gender	Compatibility, usefulness	Compatibility: No Usefulness: No
D’Onofrio et al., 2019	Pre/post design	Psychosocial measures	Ireland, Italy, and United Kingdom	Companion/social robot	PwD (*n* = 38)	Sex and gender	Well-being	No
Chen et al., 2020	RCT	Questionnaire at pre- and post-exposure	China	Social robot	PwD (*n* = 103)	Gender	Acceptance	No
Williams et al., 2021	RCT	Questionnaire, behavioral measures, interview	United States	Telehealth video-monitoring unit	IC (*n* = 42)	Gender	Attitude and usage	Attitude: No Usage: No
Xiong et al., 2020	Cross-sectional	Questionnaire	Canada	Various types to improve health/psychosocial outcomes	IC (*n* = 381)	Sex and gender	Compatibility, usage, and usefulness	Compatibility: Yes (knowledge and finances) No (installation preferences) Usage: No Usefulness: No
Sriram et al., 2021	Cross-sectional	Questionnaire	United Kingdom	Sensors, location monitoring, clocks, alarms, and reminders	IC (*n* = 201)	Sex	Well-being	Yes (mental health) No (physical health)
Wójcik et al., 2021	Cross-sectional	Questionnaire	Poland	Commercial technology (smartphones and computer)	IC (*n* = 102)	Gender	Acceptance and usage	Acceptance: Yes (smartphones) No (computer) Usage: No
Puaschitz et al., 2021	Cross-sectional	Questionnaire	Norway	Everyday technology, sensors, location monitoring, and video-communication	PwD (*n* = 276)	Sex and gender	Access	Yes (AT in general, obligatory sensors, active sensors, and everyday technology) No (passive sensors and tracking devices)
Puaschitz et al., 2023	Cross-sectional	Questionnaire, interview	Norway	Social alarm	PwD (*n* = 82) and IC (*n* = 82)	Gender	Access	No
Mishra et al., 2023	Cross-sectional	Interview, questionnaire	United States	Digital care platform	PwD (*n* = 10), IC (*n* = 14), and FC (*n* = 11)	Gender	Attitude	No

*Note.* PwD = person with dementia, IC = informal caregiver, FC = formal caregiver, RCT = randomized controlled trial, AT = assistive technology.

### Topics of Sex and Gender Analysis

The most often addressed topics of sex and gender analysis were compatibility (*n* = 5) and attitude (*n* = 4), followed by usage (*n* = 3) and usefulness (*n* = 3). Acceptance, access, and well-being were addressed to the same extent (*n* = 2, respectively). All topics, except for usage, revealed sex and gender differences. Among those, findings were mixed depending on the study or AT type.

#### Compatibility with User Values, Experiences, and Skills

Compatibility is about meeting the requirements and characteristics of the dementia care triad with AT. For PwD, ease of use depended on gender and generation for wearable location monitoring devices. The analysis of this repeated interview study with persons with mild dementia resulted in the theme of compatibility, and female PwD considered the use of AT easier for their male counterparts due to differences in contact to the technology during the upbringing of their generation (Olsson et al., 2016). Data from a survey study examined whether sex and gender was relevant in meeting individual AT needs of PwD found no significant differences (i.e., suitable conditions for AT use, *p* = .51; enabling independence by reducing external help, *p* = .35; affordability, *p* = .82; compatibility with skills, *p* = .52; and matching cultural context, *p* = .55; Asghar et al., 2019).

For informal caregivers, gender was related to previous experience and interest, which affected choice and current use of AT (Olsson et al., 2011). In this interview study, female informal caregivers were undecided toward technology due to lack of experience, yet some women reported technology knowledge and interest. Male informal caregivers reported that AT was easier to use for them due to previous experience with technology use compared to women. Data from a cross-sectional survey study exploring technology needs of informal caregivers found that the willingness to financially invest in AT and knowledge about AT was influenced by gender. Specifically, women were less willing to pay a higher amount compared to men ($501–$1000 vs. <$100: *p* = .003; >$1000 vs. <$100, *p* = .006) and had significantly more knowledge on AT than male informal caregivers (*p* = .013; Xiong et al., 2020). The same study also explored preferences regarding the installation of AT, where no differences were revealed (easy to learn to use vs. easy to install, *p* = .11; cost vs. easy to install, *p* = .21; others vs. easy to install, *p* = .91; Xiong et al., 2020).

Lastly, one questionnaire and interview study explored the perspectives of formal caregivers working in care homes. Here, female formal caregivers reported less competence for using ICT at the workplace (*p* < .01; O’Sullivan et al., 2017) with no explanation of the comparison group, assumingly male formal caregivers.

#### Attitude toward AT

The topic attitude summarizes the dementia care triad’s cognitive and affective evaluation of AT (i.e., beliefs about AT and liking or disliking AT; Yang & Yoo, 2004). Mishra et al. (2023) evaluated the attitude of the dementia care triad toward a digital care platform consisting of a wearable sensor for PwD and an accompanying caregiver app. Attitude was measured with three questionnaire items (I would use this platform daily; I would need to learn a lot of things before I could use this platform; I would feel confident using this platform) after the triad was presented with the system. Gender had no effect on attitude toward use for PwD, informal caregiver, and formal caregiver, as a short description in the discussion revealed with no comprehensible analysis presented by the paper. However, interpretation of the results is difficult without statistical parameters.

A survey study on appreciation of AT revealed that female informal caregiver appreciated tracking devices significantly more than males (*p* = .0025), while no differences were found for videoconferencing or surveillance technology (Rialle et al., 2008). Another study evaluated overall satisfaction of informal caregivers regarding a support intervention where informal caregivers received expert feedback on video recordings of challenging situations. Gender did not influence informal caregiver satisfaction with the video-monitoring unit (VMU) (it was easy to set up and use the VMU, *p* = .69; the VMU intruded on my privacy, *p* = 1.00; it was easy to capture the behavior on video, *p* = .73; having the VMU in our home was acceptable, *p* = 1.00; Williams et al., 2021).

Among formal caregivers, female employees working in care homes were less enthusiastic toward the use of ICT (*p* < .01). There was no description of who the women were compared to; however, we assume the comparison group consisted of men (O'Sullivan et al., 2017).

#### Usage

Three studies assessed the current level of use of AT by informal caregivers. No differences were revealed when informal caregivers were asked whether they had ever used AT to assist with caregiving (*p* = .54; Xiong et al., 2020). Besides, there were no sex and gender differences for the utilization of a telehealth video-monitoring intervention (Williams et al., 2021). Here, the number and duration of both phone and video submissions by informal caregivers to an expert team during the intervention was measured. Although men made more phone calls to the expert team (*M* = 10.3, *SD* = 1.5) compared to women (*M* = 8.6, *SD* = 2.7), there were no significant difference between male and female informal caregivers (number of phone calls, *p* = .064; duration of phone calls, *p* = .88; number of submitted videos, *p* = .71; duration of submitted videos, *p* = 1.00). Lastly, another study found that male and female informal caregivers equally used commercially available technology in care (i.e., smartphone, *p* = .057 and computer, *p* = .317; Wojcik et al., 2021).

#### Acceptance of AT

Acceptance is the outcome of a positive evaluation of the technology, which can be influenced by different factors, and the resulting decision by the triad to use AT in the dementia care context (Davis, 1985). For persons with dementia, Chen et al. (2020) investigated the impact of direct interaction between PwD and a social robot on changes in technology acceptance measures. In this randomized controlled trial, gender was not related to changes in technology acceptance in any of the measured domains assessed for acceptance between baseline and 32 weeks (*p* > .05 for all domains; attitude, *p* = .52; perceived usefulness, *p* = .48; perceived ease of use, *p* = .60; self-efficacy, *p* = .24, technology anxiety, *p* = .42; facilitating conditions, *p* = .51).

The acceptance of informal caregivers toward commercially available technology was also examined (Wojcik et al., 2021). Here, women were significantly more accepting toward the use of smartphones for healthcare purposes on all measures (behavioral intention, *p* = .002; social influence, *p* = .028; facilitating conditions, *p* = .034, effort expectancy, *p* = .0002; performance expectancy, *p* = .002), whereas acceptance of computer by female informal caregivers was only higher on one submeasure (behavioral intention, *p* = .018; social influence, *p* = .37; facilitating conditions, *p* = .37; effort expectancy, *p* = .07; performance expectancy, *p* = .15).

#### Access

Two studies from the same trial analyzed their data on access (i.e., whether the PwD had AT installed in the home) to AT. Baseline data from trial supporting PwD living at home and their informal caregivers revealed that AT in general (*p* = .02), everyday devices (*p* = .04), obligatory (i.e., stove guards, *p* = .01), and active sensors (i.e., social alarms, *p* = .01) were more accessible to female than to male PwD (Puaschitz et al., 2021). Access to passive sensors (e.g., fall detectors, *p* = .23) and tracking devices (*p* = .43) was equal for both male female PwD. Data from the same trial at 24 months, evaluating solely access to social alarms, found that sex and gender did neither influence access to social alarms for PwD (*p* = .99), nor for informal caregivers (*p* = .62; Puaschitz et al., 2023).

#### Usefulness

This category addresses whether AT provides sufficient support with daily functioning and care tasks judged by the dementia care triad. A cross-sectional survey study on needs and preferences of informal caregivers revealed that gender predicted perceived usefulness (*p* < .05), with women perceiving AT to be more useful than men with assisting the conduction of activities of daily life with PwD (*p* < .05; Xiong, Astell, et al., 2018). A later cross-sectional survey study addressed how informal caregivers evaluated the ability of AT to provide care and allow PwD to remain at home, where no differences on perceived usefulness were found (*p* = .099; Xiong et al., 2020).

A survey study among PwD examined the potential of AT to facilitate performance of activities of daily life. The data revealed no sex and gender differences in how AT provides physical support (e.g., mobility and physical activities; *p* = .11) and psychological support (e.g., self-confidence and encouragement; *p* = .14), or enables social interactions (*p* = .11) and traveling (*p* = .99; Asghar et al., 2019).

#### Well-Being

Well-being in its entirety is a construct determined by physical, mental, and social health (Pressman et al., 2013). Here, two studies reported on psychosocial outcomes for PwD and informal caregivers. A survey study in the United Kingdom assessed quality of life of informal caregivers who have been using AT in the previous year. Both physical and mental well-being (Short-Form Health Survey; SF-12) were assessed, where women scored significantly lower on mental health (*p* = .002) but not on physical health (*p* = .54; Sriram et al., 2021).

In addition, an international study conducted at pilot sites in Ireland, Italy, and the United Kingdom investigated the effect of interaction between PwD living in a care home and a companion robot on a variety of psychosocial outcomes where the scores did not significantly differ after interaction with the robot between male and female PwD (depression, *p* = .075; resilience, *p* = .99; quality of life, *p* = .92; perceived social support, *p* = .87; D'Onofrio et al., 2019).

### Practical Implications

Practical considerations were made by five studies, concerning development (Wojcik et al., 2021; Xiong et al., 2020), adoption (Olsson et al., 2016; Puaschitz et al., 2021; Xiong, Astell, et al., 2018; Xiong et al., 2020), policies (Puaschitz et al., 2021), advertising (Olsson et al., 2016), and daily use of AT (Olsson et al., 2011). Gender was considered to influence the daily use of AT in dementia care due to gender-related technology interest, experience, and knowledge (Olsson et al., 2011). In addition, the advertisement of AT should be tailored to gender-dependent level of interest and knowledge of technology of older generations to encourage its use (Olsson et al., 2016). Regarding development and adoption, acknowledging sex and gender differences (e.g., consider the target population; Wojcik et al., 2021) is expected to lead to improved implementation and adoption (Puaschitz et al., 2021; Wojcik et al., 2021; Xiong, Astell, et al., 2018; Xiong et al., 2020). Research on sex and gender differences is considered to be of key importance to inform healthcare services, politics, stakeholders, and governmental recommendations (Puaschitz et al., 2021).

## Discussion

This scoping review explored the role of sex and gender regarding the adoption of AT in dementia care. Herewith, we inform the areas of AT design, implementation and adoption where the consideration of sex and gender facilitates the understanding of user needs, thereby providing guidance for future clinical and research practice. Only one study explicitly focused on sex and gender as a main topic regarding AT in dementia care. The remaining studies conducted side-analyses, which illustrated that the role of sex and gender is a yet emerging topic of interest. Confirming this pattern, two-thirds of the included studies were published after 2019. Explored topics ranged from compatibility, attitude, usage, acceptance, access, usefulness, to well-being. Sex and gender differences were found for all topics, except for usage. While usage refers to actual adoption, all remaining topics can be allocated to general barriers and promotors to the adoption of AT irrespective of sex and gender (Boyle et al., 2022). The close relation of the topics to adoption highlights the practical relevance of considering sex and gender. Our review also reported areas with no sex and gender differences (e.g., equal level of current usage, physical well-being, or access to tracking technology), which is equally important so that attention can be shifted to more critical questions (Mielke et al., 2018). Practical discussions of the sex and gender-specific findings evolved around the topics of development, implementation and adoption, as well as the use of these evidence-based findings as guidelines for stakeholders (e.g., healthcare services) and governmental recommendations.

The STEM-workforce largely consists of men (Science, technology, engineering, and mathematics; majority of workforce is male; National Science Foundation, 2023). Stereotypical thinking of STEM being a male domain discourages female student’s career aspirations in science (Cundiff et al., 2013) and stereotype threats increase their likelihood to leave the field (Beasley & Fischer, 2012), thereby reinforcing gender disparities. Likewise, gender disparities regarding AT due to stereotypes could result in women benefiting from the advantages of AT (e.g., promoting independence) to a lesser extent than men. Indeed, women in dementia care were less enthusiastic about AT (O'Sullivan et al., 2017), and framed its use as typically male (Olsson et al., 2016). Gender gaps are expanded when additionally considering race (e.g., underrepresentation of black girls in STEM-programs; Joseph, 2020). Notably, this gap is also influenced by culture (i.e., narrower in more gender-equal countries; Guiso et al., 2008). Yet, lower perceived technical self-efficacy was recognized by participants from Sweden (Olsson et al., 2011), one of the relatively gender-equal countries (United Nations Development Programme, 2023). Even though PwD from Sweden hypothesized that sex and gender differences are limited to their older generation (Olsson et al., 2016), a two-third majority of German (also a relatively gender-equal country; United Nations Development Programme, 2023) high school students picture a computer scientist to be typically male and girls rate their technical self-efficacy lower than boys (Brauner et al., 2018). Following up on gender differences in perceived self-efficacy is important due to its practical consequences. First, higher perceived self-efficacy is a known facilitator of AT use in dementia care (Boyle et al., 2022) and might therefore act as a barrier for women. Second, low perceived self-efficacy of informal caregivers in dementia care negatively affects their decision to purchase a location monitoring device (Megges et al., 2017). In turn, the willingness to purchase AT is influenced by sex and gender (Xiong et al., 2020). Counteracting the barrier of self-efficacy in dementia care could follow the example of educational sciences, where positive self-efficacy is thought to encourage decision-making according to individual goals regarding higher education rather than on gender roles and stereotypes (Verdugo-Castro et al., 2022). Likewise, promoting technical self-efficacy of the dementia care triad could facilitate the adoption of AT (e.g., removing low self-efficacy as a barrier to purchase AT) according to individual goals and preferences, regardless of sex and gender or level of self-efficacy.

Another identified barrier to AT use in dementia care is lack of access (Boyle et al., 2022). This review found that female PwD more often had access to AT (Puaschitz et al., 2021), indicating an advantage for women regarding the implementation of AT (i.e., setting up AT in the home; Boyle et al., 2022). This finding is unexpected since accessing AT is more challenging for women (World Health Organization, 2022). Exploring the facilitators of implementation in female PwD’s homes could enhance implementation in dementia care regardless of sex and gender. However, equality of access varies per country (World Health Organization, 2022). Norway is considered one of the most gender-equal countries (Centre for Research on Gender Equality, 2023), which might explain both better (Puaschitz et al., 2021), and equal access for women (Puaschitz et al., 2023) in this country. In contrast, one included study reported an underrepresentation of women in their sample due to cultural norms restricting the approaching and recruiting of female PwD (Asghar et al., 2019). Hence, the privilege and equality of access to AT for women in Norway neither amend the challenge of accessing healthcare information and services for women worldwide due to culture-dependent restrictions in their mobility or decision-making power and discriminatory attitudes (World Health Organization, n.d.), nor indicate equality of access in other countries due to the lack of data within the reviewed studies. Besides, the underrepresentation of women in dementia research can have damaging effects on the development of AT (e.g., bias due to underrepresentation of data on women’s needs).

Female informal caregivers who use AT in dementia care report lower mental health than their male counterparts (Sriram et al., 2021). This finding is in line with previous research on gender disparities in mental health of caregivers, where the authors link the increased mental strain to the negative effects of caregiving which are primarily endured by women to conform with sexist beliefs associating caregiving with women (Morgan et al., 2016). In fact, women carry out the majority of informal and formal care work, which is globally undervalued, rewarded with low wages, and characterized by demanding working conditions. (World Health Organization, 2024). In addition, a shift toward informal caregiving is the consequence of chronic underinvestment in the professional healthcare sector, confronts women with an increased demand to provide unpaid caregiving as well (World Health Organization, 2024). Recognizing the urgency to address adverse mental health outcomes of informal caregivers led to the promotion of technology-based interventions, where the need to consider the recipient’s gender has also been acknowledged (Mao et al., 2023). Building on those developments, future AT should compensate for the prevailing disadvantage of female informal caregiver’s mental health, thereby allowing for equal quality of life for all users of AT.

Two of the included studies addressed the use of social robots (Chen et al., 2020; D'Onofrio et al., 2019), which are used to improve emotional, physical, and cognitive functioning of PwD by encouraging interactions and activities (Hirt et al., 2021). In both studies, sex and gender did not influence the measured outcomes after interaction with the robots (i.e., technology acceptance; Chen et al., 2020; depression, quality of life, social support, and resilience; D'Onofrio et al., 2019). A possible explanation for lack of impact of sex and gender might be that post-intervention scores did not significantly change for the entire sample except for a single outcome measure per study (perceived ease of use: *p* = .042; Chen et al., 2020; resilience: *p* = .020; D'Onofrio et al., 2019). Yet, previous research shows that sex and gender appear to be of influence regarding robots. Depending on the gender, the perception of robots differs by male and female users (Nomura, 2017). For example, females were found to have a more negative attitude than males toward the interaction with robots in a sample of college students (Nomura et al., 2006), or users of healthcare services (Kuo et al., 2009). Interestingly, not only human gender is of impact in the interaction between humans and technology, but technology can also be gendered (e.g., robots; Nomura, 2017). As is known for the application of robots, ethical issues result from people applying gender stereotypes to them (Eyssel & Hegel, 2012). Researchers warn that gender norms could subsequently be reinforced, thereby contributing to the preservation of social inequalities (Hover et al., 2021). Gender of technology was not the focus of our work, yet currently AT is being tailored to the individual users with dementia by manipulating the gender of the auditory prompts, thereby enforcing (i.e., male voice for commands) or weakening (i.e., female voice for suggestive prompts) the prompting message (König et al., 2017). Adoption of prompting technology designed in line with gender-based stereotypes (i.e., weakness associated with female gender) can likely contribute to their preservation. Awareness of the influence of sex and gender is therefore necessary as early as in the design process. Although this paper explores the role of sex and gender from the dementia care triad’s perspectives, its relevance is not limited to the perspectives of the humans who use technology, as the abovementioned examples of the gendering of robots illustrate. Therefore, fully understanding the role of sex and gender regarding AT in dementia care, consequently allowing for equitable implementation and adoption of AT in dementia care in terms of sex and gender, might require exploration from a holistic perspective (i.e., sex/ gender of the AT user and gendering of technology).

### Strengths and Limitations

Currently, the only existing research elaborating on sex/gender aspects regarding AT in dementia care has been solely from the caregiver perspective (Xiong, Astell, et al., 2018; Xiong et al., 2020; Xiong, Mansfield, et al., 2018). To our knowledge, this is therefore the first study to provide an overview of sex/gender-specific findings regarding AT adoption from the respective perspectives of the dementia care triad. Consequently, by pointing out sex/gender-specific differences for all member of the triad, this review raises awareness of the relevance to include sex and gender analysis in future studies.

A limitation of this review is that thematic inductive analysis is characterized by some level of subjectivity inherent to qualitative analysis. To address this issue, codes and categories were discussed within the research group and checked by a second author (SJ). Furthermore, both the terms sex and gender have been used by the included studies without explanation of their operationalization or interchangeably by most studies. Therefore, we lacked confidence to distinguish between the concepts. Thus, future research should commit to reporting consistent operationalizations of the terms. Besides this limitation, data was extracted only by one author (MS).

### Conclusion

The present review identifies the influence of sex and gender on the adoption of AT for the dementia care triad. Most studies not providing insight to the underlying reasons of sex and gender differences leave the question on how to customize AT open for future investigation. The included qualitative data elaborated on underlying reasons of sex and gender-specific findings and provided guidance on practical implications. Therefore, complementing quantitative data with qualitative insights will be of key importance in future research to advance adoption. Yet, the practical importance of the emerging topics has been established due to their close relation to the concept of adoption. Adoption might also differ per type of AT, since the influence of sex and gender on the analyzed topics were divergent depending on both type of AT or the respective studies. Thus, accommodating multiple types of AT in a single analysis might not sufficiently acknowledge their heterogeneity. Establishing standard categories of AT in research and practice could enhance our understanding of the different types of AT. In addition, the findings (e.g., stereotypes and underrepresentation of women in research) surpass the context of dementia care and AT, proving their broader relevance in society. These issues, potentially acting as barriers to the design, implementation, and adoption of AT, should be addressed (i.e., disaggregating of data by sex and gender in research) with priority in the predominantly female user population in dementia care. Thereby, knowledge on the role of sex and gender in AT adoption can be broadened and result in meeting the needs of the dementia care triad more efficiently.

## Supplemental Material

Supplemental Material - Exploring the Role of Sex and Gender in the Adoption of Assistive Technology in Dementia Care: A Scoping Review Supplemental Material for Exploring the Role of Sex and Gender in the Adoption of Assistive Technology in Dementia Care: A Scoping Review by Maren Salzwedel, Sytse Zuidema, Helianthe Kort, and Sarah Janus in Journal of Applied Gerontology.
