# Supplementary material for: Exploring the Role of Sex and Gender in the Adoption of Assistive Technology in Dementia Care: A Scoping Review
Source: J Appl Gerontol. 2025 Jan 9;44(9):1458–71. doi: 10.1177/07334648241310708 (PMC12335632; doi:10.1177/07334648241310708)
Supplement: Supplemental Material - Exploring the Role of Sex and Gender in the Adoption of Assistive Technology in Dementia Care: A Scoping Review [file sj-pdf-1-jag-10.1177_07334648241310708.pdf]

## Appendix A

Search string PubMed

("Dementia"[Mesh] OR dementia*[ti] OR Alzheimer*[ti] OR "lewy body disease*"[ti])

AND

("Technology"[Mesh] OR technol*[ti] OR telecare[ti] OR ATT[ti] OR "Self-Help Devices"[Mesh:NoExp] OR "assistive device*"[ti] OR "assistive technol*"[ti] OR "Telecommunications"[Mesh] OR telemedicine[ti] OR "mobile health"[ti] OR mhealth[ti] OR surveillance[ti] OR tagging[ti] OR tag[ti] OR track*[ti] OR monitor*[ti] OR position*[ti] OR reminder*[ti] OR prompt*[ti] OR alert*[ti] OR alarm*[ti] OR "communication aids"[ti] OR "communication aid"[ti] OR "Wearable Electronic Devices"[Mesh:NoExp] OR Wearable*[ti] OR smartphone*[ti] OR "Mobile Applications"[Mesh] OR "mobile applicat*"[ti] OR "mobile technol*"[ti] OR sensor*[ti] OR smarthome*[ti] OR "smart home*"[ti] OR wireless[ti] OR "Information Technology"[Mesh] OR "internet of things"[ti] OR iot[ti] OR gps[ti] OR robot*[ti] OR digital[ti])

AND

("Sex Characteristics"[Mesh] OR "Gender Role"[Mesh] OR "Sex"[Mesh] OR sex[tiab] OR gender*[tiab] OR female*[tiab] OR male*[tiab] OR men[tiab] OR woman[tiab] OR women[tiab])

**Appendix B**

*Thematic inductive analysis*

| Study | Data unit | Summary data unit | Code | Categories | Definition of Categories |
|---|---|---|---|---|---|
| Williams et al., 2021 | Relationship between gender (male/female) of IC and satisfaction VMU<br>- It was easy to set up and use the VMU (*p*= .694)<br>- The VMU intruded on my privacy (*p*= 1.000)<br>- It was easy to capture the behavior on video (*p*= .729)<br>- Having the VMU in our home was acceptable (*p*= 1.000) | Women and men were equally satisfied with intervention (privacy, ease of use and set-up, acceptability) | Satisfaction of IC with telehealth intervention | Attitude | The dementia care triad's feelings towards the use of AT (cognitive and affective evaluation of AT such as beliefs or liking) |
| Mishra et al., 2023 | Effect of gender on attitude towards technology and intention to use of sample (PwD, IC, FC) when being presented to care platform<br>- I would use this platform daily<br>- I would need to learn a lot of things before I could use this platform<br>- I would feel confident using this platform | Gender had no effect on attitude | Attitude of triad towards care platform | Attitude | |
| O'Sullivan et al., 2017 | Results showed that older and female professional caregivers working at nursing homes, respectively, reported less enthusiasm (z = -2.38, p < .05 | Female professional caregivers report less enthusiasm regarding ICT use at workplace | Attitude of FC towards technology use in care homes for FC | Attitude | |

| Study | Data unit | Summary data unit | Code | Categories | Definition of Categories |
|---|---|---|---|---|---|
| | and z = -4.01, p < .01, resp.) regarding the use of technology in their work environment. | | | | |
| Rialle et al., 2008 | The most appreciated technology, the tracking device, with female caregivers appreciating it significantly more than male (p = .0025, N=195). The second most appreciated technology was the videoconferencing device for social connectedness with no significant difference between male and female caregivers. The least rejected device was the personal pocket videoconferencing device for mobile private remote surveillance of the patient, with also no significant difference between male and female caregivers. | Differences between male and female IC in appreciation for tracking devices, not for videoconferencing devices or mobile surveillance device | Attitude of IC towards tracking devices, videoconferencing devices, remote surveillance | Attitude | |
| Chen et al., 2020 | Influence of gender not significant on technology acceptance post intervention (direct interaction with humanoid social robot, RCT of 32 weeks) for any domain measured by senior technology acceptance model per domain: p > 0.05 for all domains; attitude: $p$= .52; perceived usefulness: $p$= .48; perceived ease of use: $p$= .60; self-efficacy: $p$= .24, technology anxiety: $p$= .42; facilitating conditions: $p$= .51 | Gender has no impact on technology acceptance of PwD measured by attitude, perceived usefulness, perceived ease of use, technology anxiety and facilitating conditions | Acceptance | Acceptance of AT | Acceptance is the outcome of a positive evaluation of the technology, which can be influenced by different factors, and the resulting decision by the triad to use AT in the dementia care context |

| Study | Data unit | Summary data unit | Code | Categories | Definition of Categories |
|---|---|---|---|---|---|
| Wójcik et al., 2021 | Acceptance of commercially available technology used for care purposes as measured by a to health care devices adapted version of UTAUT:<br>- Acceptance of use of smartphones: behavioral intention: *p*= .0015*; social influence: *p*= .0277*; facilitating conditions: *p*= .0344*, effort expectancy: *p*= .0002*; performance expectancy: *p*= .0020*)<br>- Acceptance of use of computers: behavioral intention: *p*= .0183*, social influence: *p*= .3668; facilitating conditions: *p*= .3703; effort expectancy: *p*= .0698; performance expectancy: *p*= .1514<br>*female IC scored higher than male IC | Female IC were more accepting towards smartphone use in care compared to males; for computer use acceptance was only higher for females on one acceptance component | Acceptance | Acceptance of AT | |
| Xiong et al., 2018 | Female IC found technology to be significantly more useful in helping with the care recipient's ADLs when compared to their male counterparts (Wilcoxon Signed Ranks Test. Z = -2.21, p < 0.05)<br>Multiple regression analysis revealed that sex and gender was a significant | - Female IC found technology more useful than males for assistance with ADL<br>- Sex/gender predicted perceived | Perceived usefulness of AT | Usefulness of AT | Addresses whether AT provides sufficient support with daily functioning and care tasks judged by the dementia care triad. |

| Study | Data unit | Summary data unit | Code | Categories | Definition of Categories |
|---|---|---|---|---|---|
| | predictor of perceived usefulness of technology (ß= 1.25, p < 0.05). | usefulness | | | |
| Xiong et al., 2020 | Perceived usefulness of IC regarding technology: Sex and gender (ref: male): ß (95% CI)= 0.16 (−0.17–0.49), *p*= .099 | No gender differences on perceived usefulness of technology of IC | Perceived usefulness | Usefulness of AT | |
| Asghar et al., 2019 | Factor analysis: Gender had no influence on factors; Mann-Whitney U Test (Male and Female): physical support: *p*= .109; psychological support: *p*= .141; social support: *p*= .109;<br>travel help: *p*= .988 | Gender had no influence on factors determining support of AT with conduction of daily activities (i.e., assisting with mobility and physical performance of daily activities, increasing self-esteem, enabling social interaction and traveling) | Perceived support with conduction of daily activities | Usefulness of AT | |
| Sriram et al., 2021 | General physical (PCS) and mental (MCS) wellbeing of IC who used AT for previous year:<br>Women score significantly lower on MCS compared to men (p = .002), but not on PCS (*p*= .536) | Female IC using AT for previous year score lower on mental wellbeing than men, no difference n physical wellbeing | General physical and mental well-being of persons who use AT | Well-being of AT users | Well-being in its entirety is a construct determined by physical, mental and social health. |
| D'Onofrio et al., 2019 | Depression, resilience, QoL, perceived social support measures post MARIO robot for PwD (all p > .05: CSDD: *p*= 0.075; RS-14: *p*= .987; QoL-AD: *p*= .919; MSPSS Total: *p*= .872; 51.<br>*only resilience improved after | No gender differences in PwD on depression, resilience, QoL, perceived social support after social robot intervention | Well – being (QoL, mental health, resilience, social support) of PwD | Well-being of AT users | |

| Study | Data unit | Summary data unit | Code | Categories | Definition of Categories |
|---|---|---|---|---|---|
| | MARIO intervention for whole group | | | | |
| Puaschitz et al., 2021 | Access more often available for female PwD compared to male: AT in general (*p*= .02), everyday devices (*p*= .04), obligatory (i.e., stove guards; *p*= .01), active sensors (i.e., social alarms; *p*= .01)<br>Equal access for male and female PwD: passive sensors (e.g., fall detectors, *p*= .23), tracking devices (*p*= .43). | Gender as an associated factor with access depending on AT subtype, access equal or better for women | Access to AT for PwD | Access to AT | Whether the PwD had AT installed in the home |
| Puaschitz et al., 2023 | Factors associated with access to social alarms among 82 home-living people with dementia at 24 months:<br>PwD: Gender, male: OR= 1.01 , CI (0.26–3.88) , *p*= .99<br>IC: Gender, male: OR= 0.61 , CI (0.09–4.30) , *p*= .62 | Gender no associated factor with access to AT for PwD and IC | Access to AT for PwD and IC | Access to AT | |
| Xiong et al., 2020 | Current level of use of technology by IC: Sex and gender (ref: male): OR (95% CI) = 0.73 (0.27–1.97), *p*= .54 | No gender difference in current technology use for IC | Level of current technology use by IC | Usage of AT | Current level of usage of AT |
| Wójcik et al., 2021 | In dementia care use of commercially available technology by IC:<br>- Current smartphone use: *p*= .0568<br>- Current computer use: *p*= .3169 | No gender differences in current smartphone or computer use | Level of current technology use by IC | Usage of AT | |

| Study | Data unit | Summary data unit | Code | Categories | Definition of Categories |
|---|---|---|---|---|---|
| Williams et al., 2012 | Relationship between utilization and gender (male/female) of VMU for IC:<br>- Number of phone calls ($p$= .064)<br>- Duration of phone calls ($p$= .879)<br>- Number of videos ($p$= .709)<br>- Duration of videos ($p$= 1.000 | Women and men used the VMU to the same extent | Extent to which VMU was used by IC | Usage of AT | |
| Xiong et al., 2020 | Cost of technology: Sex and gender (ref: male):<br>- \$100 - \$500 vs < \$100: OR (95% CI)= 0.60 (0.29–1.22), $p$= .16<br>- \$501 -\$1000 vs < \$100: OR (95% CI)= 0.27 (0.11–0.64), $p$= .0031<br>- > \$1000 vs < \$100: OR (95% CI)= 0.21 (0.070–0.64), $p$= .005 | Male IC were willing to pay higher amounts | Compatibility with financial values | Compatibility with values, experiences, skills | Meeting the requirements and characteristics of the dementia care triad with AT |
| Xiong et al., 2020 | Feature preferences when setting up technology<br>- Sex and gender (ref: male): Easy to learn to use vs easy to install OR (95% CI)= 0.56 (0.28–1.14), $p$ =.11<br>- Cost vs easy to install OR (95% CI)= 1.69 (0.74–3.86), $p$= .21<br>- Others vs easy to install: OR (95% CI)= 1.06 (0.39–2.89), $p$= .91 | No gender differences on feature preferences setting up technology | Compatibility with user values for installation | Compatibility with user of AT | |

| Study | Data unit | Summary data unit | Code | Categories | Definition of Categories |
|---|---|---|---|---|---|
| Xiong et al., 2020 | Knowledge of technology: Sex and gender (ref: male): OR (95% CI)= 3.93 (1.33–11.63), *p*= .013 | Female IC had more knowledge on care technology | Compatibility with users level of knowledge | Compatibility with users of AT | |
| O'Sullivan et al., 2017 | Results showed that older and female professional caregivers working at nursing homes, respectively, reported less competence (z = -4.21, p < .01 and z = -4.44, p < .01, resp.) regarding the use of technology in their work environment. | Female professional caregivers report less competence regarding ICT use at workplace | Compatibility with FC level of competence | Compatibility with users of AT | |
| Olsson et al., 2016 | Compatibility (Consistency of technology with values, experiences, and needs of technology users) with technology according to PwD: "Just in general they [men] are more technology freaks than we women are, especially in this generation, but for the next generation it will be different because both sexes have grown up with technology. Then I thought, yeah, typically male, a little 'calm security'". One female participant thought it might be easier to get men interested in starting to use the PPA: "If they [men] get something technical and it's explained as something technical, then that changes things, then there's status involved or … I think it could work". Another female participant | Female PwD think men are more interested and familiar in technology, especially for older generations, which could serve as an advantage when interesting men in using technology and during use of technology | Compatibility with interest and experience of PwD due to generation | Compatibility with users of AT | |

| Study | Data unit | Summary data unit | Code | Categories | Definition of Categories |
|---|---|---|---|---|---|
| | said: “I mean men like machines so it shouldn’t be a problem, I don’t think, giving them one, getting them to think they’ve become technical, then it will work”. | | | | |
| Olsson et al., 2011 | Knowledge, skills and abilities regarding ICT use in dementia care according to IC:<br><br>Female informants found it difficult to know what kind of ICT to use, and they said it was their husband (the person with dementia) who had previously taken care of technological issues. However, some female informants had ICT knowledge and skills based on their own interest or previous occupation. One male informant described a gender perspective in the following way: It seems to me it might be harder in many situations... deciding what technical solutions I should come up with at home // which is usually easier for a man who uses technology more often // it’s not easy for all men either, but it is more common for us to have screwdrivers and wrenches at home. | Gender influences ease of decision about what type of ICT to use because husbands dealt with technology, some women reported skills and knowledge due to occupation | Compatibility with experience, knowledge and skills of IC | Compatibility with users of AT | |
| Asghar et al., 2019 | Factor analysis: Gender had no influence on factors; Mann-Whitney U Test (Male and Female): | Gender does not influence factors determining the match between AT and | Matching of AT with the dementia care triad | Compatibility of AT with users | |

| Study | Data unit | Summary data unit | Code | Categories | Definition of Categories |
|---|---|---|---|---|---|
| | operational support: *p*= .508; cultural match: *p*= .552; reduced external help: *p*= .353; affordability: *p*= .822; compatibility: *p*= .522 | users in terms of conditions of AT use, culture, ability to use AT independently, culture, affordability, of general abilities | | | |

*Note*. Code = essence of meaning of data units; Category = grouping of codes according to common theme; AT = Assistive technology; CI = Confidence interval; OR = Odds ratio; CSDD = Cornell Scale for Depression in Dementia, RS-14 = 14item Resilience Scale, QoL-AD = Quality of Life in Alzheimer's Disease, MSPSS = Multidimensional Scale of Perceived Social Support; PwD = Person with dementia, IC = Informal caregiver, FC = formal caregiver; VMU = telehealth video monitoring unit; RCT = Randomized controlled trial; UTAUT = Unified theory of technology acceptance; ICT = Information and communications technology

# Appendix C

## The role of sex and gender regarding assistive technology in dementia care: A scoping review protocol

According to the World Health Organization (WHO; 2023), 55 million people are currently living with dementia and this number is increasing by 10 million new cases each year. In order to alleviate the rising burden of dementia, assistive technology (AT) is increasingly promoted in dementia care (Gathercole et al., 2021; Lariviere et al., 2021). AT use in dementia care aims to support persons living with dementia (PwD) and their informal (e.g., friends, family) and formal caregivers (Gibson et al., 2015, Hall et al. 2017). Purposes of use range from encouraging the PwD's independence (Holthe et al., 2022; Gathercole et al., 2021; Lariviere et al., 2021), supporting meaningful activities during leisure time (Gathercole et al., 2021), increasing social participation, improving both PwD's and caregiver's quality of life, reducing caregiver burden for both informal and formal caregiver and healthcare costs, managing behavioral symptoms of PwD (Dugstad et al., 2019; Lee-Cheong et al., 2022), to improving safety both in the home setting (Holthe et al., 2022; Lee-Cheong et al., 2022) and in care homes (Dugstad et al., 2019; Hall et al., 2017).

Generally, the perception and acceptance of technology is moderated by particular factors, including gender (Heerink, 2011). Yet, research on dementia care has not focused on the role of sex and gender aspects regarding technology (Castro-Aldrete; Xiong et al.,2020). Still, there is a small amount of research suggesting that the acceptance of technologies in dementia care is influenced by gender from the informal caregivers' perspective before implementation (Hvalič-Touzery et al., 2021). Furthermore, male, and female spouses of PwD approach the use of AT differently (e.g., with different beliefs of their capability to use AT; Olsson et al., 2013; 2016). Specifically, female PwD think that using AT is easier for male PwD than for themselves (Olsson et al., 2016). Also, female spouses of PwD questioned their own capability of correctly using AT more frequently than their male counterparts (Olsson et al., 2013). Still, female caregivers can perceive the use of AT as more useful than men. (Xiong et al., 2018). Notably, perceived usefulness determines valuable implementation and adoption of AT (Boyle et al., 2022; van Boekel et al., 2019; Sriram et al., 2019). In addition to that, numerous barriers have been identified in the implementation and adoption of AT (e.g., costs, limited awareness; Boyle et al., 2022; knowledgeability; Sriram et al., 2019; Xiong et al., 2020; finances (Xiong et al. 2020). Despite the role of sex and gender regarding AT in dementia care, this perspective on technology has not been elaborated in detail yet (Xiong et al., 2020).

Including a sex and gender perspective on AT is especially relevant in the dementia care setting, where women are disproportionately affected by the disease (WHO; 2023) and are more likely to provide care to a PwD (Derreberry and Holroyd, 2019; Mao et al., 2021). However, females are less likely than males to access AT (WHO, 2022), yet they appear to be the main users in dementia care. Notably, AT is not always gender friendly (WHO, 2022). Research suggests incorporating sex and

gender factors as a standard practice when examining intervention implementation and their barriers (Tannenbaum et al., 2016). Sex and gender influences communication, decision-making or the uptake of interventions (Tannenbaum et al., 2016). Attention is drawn to the importance of examining sex and gender aspects in the development of technologies that will likely be used for decades (Castro-Aldrete et al., 2023). Therefore, this scoping review will elaborate on the current role of sex and gender aspects regarding AT in dementia care, specifically

1. What are the topics that are analyzed in terms of sex and gender?

2. What are the sex- and gender specific findings? For whom?

3. Is there a discussion of the practical implications, if yes: what are the conclusions?

## Method

This scoping review followed the methodological framework of Arksey and O'Malley (2005), with additional guidance by Peters et al. (2022).

### Eligibility criteria

The research questions were developed based on the PCC mnemonic, which outlines the population, concept, and context of the review (Peters et al., 2022). The following outline has been developed: (P) dementia care triad (i.e., PwD, informal caregiver, professional caregiver), (C) role of sex and gender factors (C) AT in dementia care setting (community/home setting, care home setting).

Based on this mnemonic the following in- and exclusion criteria have been formulated Inclusion criteria: (1) AT in line with the definition of the review, (2) used by dementia care triad, (3) disaggregation of data by sex or gender, (4) peer-reviewed articles and conference papers, (5) primary studies, and (6) published in English, Dutch or German. Studies that targeted AT (1) in the context of mild cognitive impairment, (2) for therapeutic purposes, or (3) diagnostic purposes, or (4) only reporting the sex and gender distribution of the sample without analysis, (5) or mixed samples were excluded.

### Information Sources

The chosen databases were PubMed, IEEE, and Web of Science with an additional consultation of the grey literature (i.e., conference abstracts, Google Scholar).

### Search Strategy

The set up of the strategy involved the use of Boolean search operators (i.e., AND, OR) and consisted of search terms representing the topics "assistive technology", "sex or gender" and "dementia". The search strategy was defined and revised in accordance with the advice of a medical information specialist at the Central Medical Library of the University Medical Center Groningen.

The complete search string contains the following terms:

("Dementia"[Mesh] OR dementia*[ti] OR Alzheimer*[ti] OR "lewy body disease*"[ti])

AND

("Technology"[Mesh] OR technol*[ti] OR telecare[ti] OR ATT[ti] OR "Self-Help Devices"[Mesh:NoExp] OR "assistive device*"[ti] OR "assistive technol*"[ti] OR "Telecommunications"[Mesh] OR telemedicine[ti] OR "mobile health"[ti] OR mhealth[ti] OR surveillance[ti] OR tagging[ti] OR tag[ti] OR track*[ti] OR monitor*[ti] OR position*[ti] OR reminder*[ti] OR prompt*[ti] OR alert*[ti] OR alarm*[ti] OR "communication aids"[ti] OR "communication aid"[ti] OR "Wearable Electronic Devices"[Mesh:NoExp] OR Wearable*[ti] OR smartphone*[ti] OR "Mobile Applications"[Mesh] OR "mobile applicat*"[ti] OR "mobile technol*"[ti] OR sensor*[ti] OR smarthome*[ti] OR "smart home*"[ti] OR wireless[ti] OR "Information Technology"[Mesh] OR "internet of things"[ti] OR iot[ti] OR gps[ti] OR robot*[ti] OR digital[ti])

AND

("Sex Characteristics"[Mesh] OR "Gender Role"[Mesh] OR "Sex"[Mesh] OR sex[tiab] OR gender*[tiab] OR female*[tiab] OR male*[tiab] OR men[tiab] OR woman[tiab] OR women[tiab]).

**Selection process**

The identified studies will be imported to the reference manager EndNote for manual duplicate removal. The remaining results will be imported into the Rayyan screening tool for a screening of the titles and abstracts. The titles and abstracts will be screened by two authors (SJ, MS), assuring mutual independence by using the blinding setting. The articles identified as eligible by title and abstract will undergo a full-text screening.

**Data collection process**

The data of the studies included in the review by full-text screening will be charted in an Excel spreadsheet by one author (MS). Information will be collected on the topics of sex- and gender analysis, sex- and gender specific findings and their practical implications, type of AT and its intended function.

**Data charting**

The following table will be used for charting the data.

| Study | Type of study | Type of technology/device | AT function | Purpose of the study | Findings of study | Sex/gender distribution of sample | Member of triad | Topic of analysis | Sex- and gender specific findings | Practical implications |
|---|---|---|---|---|---|---|---|---|---|---|
| … | … | … | … | … | … | … | … | … | … | … |

**Collating, summarizing, and reporting the results**

Data on the above defined outcomes will be presented in table form alongside a narrative description of the results. Here, we will identify for which topics sex and gender factors have been analyzed and whether they play role in the use of AT (i.e., are there differences, what practical implications do they have).

**Consultation Exercise**

In advance of the search the client panel of the university network for elderly care of the University Medical Center Groningen will be consulted about our search strategy. Members will be asked to give feedback on the search terms and add their perspectives to it. The search strategy will be adjusted accordingly (i.e., search terms relevant to the panel were added) and used for the search.


## Funding

This research is conducted within the SPREAD+ consortium as part of the ZonMW Dementia Research Program, and financially supported by Alzheimer Nederland and Health Holland (WE.32-2022- 01).


## Conflict of interest

We have no conflicts of interest to declare.

## Note

This protocol was guided by the best practice of Joanna Briggs institute (Peters et al, 2022) and registered with the Open Science Framework (https://doi.org/10.17605/OSF.IO/8VKZJ).

## References

Arksey, H., & O'Malley, L. (2005). Scoping studies: Towards a methodological framework. *International Journal of Social Research Methodology*, *8*(1), 19–32. https://doi.org/10.1080/1364557032000119616

Boyle, L. D., Husebo, B. S., & Vislapuu, M. (2022). Promotors and barriers to the implementation and adoption of assistive technology and telecare for people with dementia and their caregivers: A systematic review of the literature. *BMC Health Services Research*, *22*(1), 1573. https://doi.org/10.1186/s12913-022-08968-2

Castro-Aldrete, L., Moser, M. V., Putignano, G., Ferretti, M. T., Schumacher Dimech, A., & Santuccione Chadha, A. (2023). Sex and gender considerations in Alzheimer’s disease: The Women's Brain Project contribution. *Frontiers in Aging Neuroscience*, *15*, 1105620–1105620. https://doi.org/10.3389/fnagi.2023.1105620

Daly-Lynn, J., Ryan, A., McCormack, B., & Martin, S. (2023). Stakeholder's experiences of living and caring in technology-rich supported living environments for tenants living with dementia. *BMC Geriatrics*, *23*(1), 62. https://doi.org/10.1186/s12877-023-03751-2

Derreberry, T. M., & Holroyd, S. (2019). Dementia in Women. *The Medical clinics of North America*, *103*(4), 713–721. https://doi.org/10.1016/j.mcna.2019.02.004

Dugstad, J., Eide, T., Nilsen, E. R., & Eide, H. (2019). Towards successful digital transformation through co-creation: A longitudinal study of a four-year implementation of digital monitoring technology in residential care for persons with dementia. *BMC Health Services Research*, *19*(1), 366. https://doi.org/10.1186/s12913-019-4191-1

Gathercole, R., Bradley, R., Harper, E., Davies, L., Pank, L., Lam, N., Davies, A., Talbot, E., Hooper, E., Winson, R., Scutt, B., Montano, V. O., Nunn, S., Lavelle, G., Lariviere, M., Hirani, S., Brini, S., Bateman, A., Bentham, P., … Howard, R. (2021). Assistive technology and telecare to maintain independent living at home for people with dementia: The ATTILA RCT. *Health Technology Assessment*, *25*(19), 1–156. https://doi.org/10.3310/hta25190

Gibson, G., Dickinson, C., Brittain, K., & Robinson, L. (2015). The everyday use of assistive technology by people with dementia and their family carers: A qualitative study. *BMC Geriatrics*, *15*, 89. https://doi.org/10.1186/s12877-015-0091-3

Hall, A., Wilson, C. B., Stanmore, E., & Todd, C. (2017). Implementing monitoring technologies in care homes for people with dementia: A qualitative exploration using Normalization Process Theory. *International Journal of Nursing Studies*, *72*, 60–70. https://doi.org/10.1016/j.ijnurstu.2017.04.008

Heerink, Marcel. (2011). *Exploring the influence of age, gender, education and computer experience on robot acceptance by older adults* [Conference presentation abstract]. HRI 2011 - Proceedings of the 6th ACM/IEEE International Conference on Human-Robot Interaction. 147-148. 10.1145/1957656.1957704.

Hvalič-Touzery, S., Dolničar, V., & Prevodnik, K. (2022). Factors influencing informal carers' acceptance of assistive telecare systems in the pre- and post-implementation phase: A scoping study. *Health & Social Care in the Community*, *30*(5), e1484–e1504. https://doi.org/10.1111/hsc.13840

Holthe, T., Halvorsrud, L., & Lund, A. (2022). Digital Assistive Technology to Support Everyday Living in Community-Dwelling Older Adults with Mild Cognitive Impairment and Dementia. *Clinical Interventions in Aging*, *17*, 519–544. https://doi.org/10.2147/CIA.S357860)

Lariviere, M., Poland, F., Woolham, J., Newman, S., & Fox, C. (2021). Placing assistive technology and telecare in everyday practices of people with dementia and their caregivers: Findings from an embedded ethnography of a national dementia trial. *BMC Geriatrics*, *21*(1), 121. https://doi.org/10.1186/s12877-020-01896-y

Lee-Cheong, S., Amanullah, S., & Jardine, M. (2022). New assistive technologies in dementia and mild cognitive impairment care: A PubMed review. *Asian Journal of Psychiatry*, *73*, 103135. https://doi.org/10.1016/j.ajp.2022.103135

Mao, A., Cheong, P. L., Van, I. K., & Tam, H. L. (2021). “I am called girl, but that doesn’t matter” - Perspectives of male nurses regarding gender-related advantages and disadvantages in professional development. *BMC Nursing*, *20*(1). https://doi.org/10.1186/s12912-021-00539-w

Olsson, A., Engström, M., Lampic, C., & Skovdahl, K. (2013). A passive positioning alarm used by persons with dementia and their spouses - A qualitative intervention study. *BMC Geriatrics*, *13*, 11. https://doi.org/10.1186/1471-2318-13-11

Olsson, A., Skovdahl, K., & Engström, M. (2016). Using diffusion of innovation theory to describe perceptions of a passive positioning alarm among persons with mild dementia: A repeated interview study. *BMC Geriatrics*, *16,* 3. https://doi.org/10.1186/s12877-016-0183-8

Peters, M. D. J., Godfrey, C., McInerney, P., Khalil, H., Larsen, P., Marnie, C., Pollock, D., Tricco, A. C., & Munn, Z. (2022). Best practice guidance and reporting items for the development of scoping review protocols. *JBI Evidence Synthesis*, *20*(4), 953–968. https://doi.org/10.11124/JBIES-21-00242

Sriram, V., Jenkinson, C., & Peters, M. (2019). Informal carers' experience of assistive technology use in dementia care at home: A systematic review. *BMC Geriatrics*, *19*(1), 160. https://doi.org/10.1186/s12877-019-1169-0

Tannenbaum, C., Greaves, L., & Graham, I. D. (2016). Why sex and gender matter in implementation research. *BMC Medical Research Methodology*, *16*(1), 145. https://doi.org/10.1186/s12874-016-0247-7

World Health Organization and the United Nations Children's Fund (2022). *Global report on assistive technology*.

World Health Organization. (2023, March 15). *Dementia.* Retrieved 5 June 2023 from https://www.who.int/news-room/fact-sheets/detail/dementia

Xiong, C., Astell, A., Mihailidis, A., & Colantonio, A. (2018). Needs and preferences for technology among Chinese family caregivers of persons with dementia: A pilot study. *Journal of Rehabilitation and Assistive Technologies Engineering*, *5*, 2055668318775315. https://doi.org/10.1177/2055668318775315

Xiong, C., Ye, B., Mihailidis, A., Cameron, J. I., Astell, A., Nalder, E., & Colantonio, A. (2020). Sex and gender differences in technology needs and preferences among informal caregivers of persons with dementia. *BMC Geriatrics*, *20*(1), 176. https://doi.org/10.1186/s12877-020-01548-1
